# *Bifidobacterium animalis* subsp. *lactis* TISTR 2591 Improves Glycemic Control and Immune Response in Adults with Type 2 Diabetes Mellitus: A Randomized, Double-Blind, Placebo-Controlled Crossover Clinical Trial

**DOI:** 10.3390/nu17193097

**Published:** 2025-09-29

**Authors:** Wiritphon Khiaolaongam, Kongsak Boonyapranai, Jaruwan Sitdhipol, Punnathorn Thaveethaptaikul, Jurairat Khongrum, Pennapa Chonpathompikunlert, Sakaewan Ounjaijean

**Affiliations:** 1 School of Health Sciences Research, Research Institute for Health Sciences, Chiang Mai University, Chiang Mai 50200, Thailand; wiritphon_k@cmu.ac.th; 2 Research Institute for Health Sciences, Chiang Mai University, Chiang Mai 50200, Thailand; kongsak.b@cmu.ac.th; 3 Biodiversity Research Centre, Thailand Institute of Scientific and Technological Research (TISTR), Pathumtani 12120, Thailand; jaruwan_s@tistr.or.th (J.S.); punnathorn@tistr.or.th (P.T.); 4 Multidisciplinary Research Institute, Chiang Mai University, Chiang Mai 50200, Thailand; jurairat.kh@cmu.ac.th

**Keywords:** probiotics, *Bifidobacterium animalis* subsp. *lactis* TISTR 2591, glycemic control, immune response, type 2 diabetes mellitus, randomized controlled trial

## Abstract

**Background/Objectives**: Type 2 diabetes mellitus (T2DM) is a chronic metabolic disorder marked by insulin resistance, hyperglycemia, systemic inflammation, and immune imbalance. This randomized, double-blind, placebo-controlled, crossover trial investigated the effects of *Bifidobacterium animalis* subsp. *lactis* TISTR 2591 (BA-2591), a probiotic strain isolated in Thailand, on metabolic, immunologic, and safety parameters. **Methods**: A total of 44 Thai adults (aged 35–65) with T2DM receiving metformin monotherapy were administered BA-2591 (1 × 10^9^ CFU/g/day) or placebo for 6 weeks, followed by a 4-week washout and crossover. **Results**: Compared to placebo, BA-2591 significantly attenuated fasting blood glucose elevation (Δ = +1.143 mg/dL vs. +12.570 mg/dL; *p* < 0.001), minimized the increase in insulin resistance (HOMA-IR: Δ = +0.567 vs. +0.980; *p* = 0.006), and enhanced β-cell function (HOMA-β: Δ = +6.791% vs. −8.313%; *p* < 0.001). It also elevated immunoglobulin levels (IgM: +150.300 mg/dL; IgG: +261.500 mg/dL; *p* < 0.001), reduced LDL-C (*p* = 0.009), and decreased cathepsin D activity (*p* = 0.005), with no significant changes in IL-6, adiponectin, MDA, hs-CRP, or body composition. No severe adverse effects were reported. **Conclusions**: BA-2591 was safe and demonstrated modest, adjunctive benefits for fasting glycemia and immunologic profiles over 6 weeks, without changes in body weight or fat mass. These findings support BA-2591 as a potential adjunct to standard care in early T2DM; larger and longer-duration trials are needed to define its effects on longer-term outcomes.

## 1. Introduction

Type 2 diabetes mellitus (T2DM), a chronic metabolic disorder characterized by insulin resistance, β-cell dysfunction, and elevated blood glucose, represents a significant and growing public health burden worldwide. The global prevalence of T2DM is projected to affect nearly 700 million adults by 2045 [1]. Alongside glycemic dysregulation, patients with T2DM frequently exhibit co-morbidities such as dyslipidemia, systemic inflammation, oxidative stress, and compromised immune function, all of which contribute to serious microvascular and macrovascular complications, including nephropathy, retinopathy, and cardiovascular disease [2,3].

Current conventional treatment strategies for T2DM primarily involve pharmacological agents such as metformin, sulfonylureas, or insulin therapy. While effective, these approaches are often associated with undesirable side effects including gastrointestinal discomfort, hypoglycemia, and long-term metabolic consequences [4]. This has stimulated a growing interest in adjunctive interventions, particularly those targeting the gut microbiome, which has emerged as a pivotal regulator of host metabolism, immune function, and glucose homeostasis [5].

Recent research underscores the potential of probiotics—live microorganisms that confer health benefits on the host—as metabolic modulators in T2DM. Among these, *Bifidobacterium* spp. has drawn particular attention for its ability to modulate gut microbial composition, enhance short-chain fatty acid (SCFA) production, and reduce intestinal permeability and inflammation [3,6]. Moreover, clinical trials involving *Bifidobacterium* strains have reported improvements in lipid profile, glycemic markers such as HbA1c, and systemic oxidative stress [7].

*Bifidobacterium animalis* subsp. *lactis* TISTR 2591 (BA-2591), a Thai-derived bacterial strain, has demonstrated favorable probiotic properties including high adhesion ability, acid-bile tolerance, and antimicrobial activity against enteropathogens [8]. Preclinical studies have shown that supplementation with *B. animalis* improves insulin sensitivity, lowers fasting blood glucose levels and enhances hepatic antioxidant defense in rodent models of diabetes [9,10]. These findings are further substantiated by evidence indicating that probiotic interventions can modulate immune responses by upregulating anti-inflammatory cytokines like IL-10 and boosting immunoglobulin levels [4,8].

Despite this promise, the strain-specific efficacy and safety of BA-2591 in human diabetic populations remains underexplored. While general evidence supports the use of probiotics in metabolic disorders, the high heterogeneity of probiotic strains, dosing regimens, and patient characteristics necessitate precise clinical validation [7]. Given that BA2591 is indigenous to the Thai population and demonstrates strong gastrointestinal resilience, it stands as a candidate of interest for localized therapeutic application [8]. This study aims to investigate the metabolic, immunological, and oxidative impacts of BA-2591 supplementation in Thai adults with recently diagnosed type 2 diabetes mellitus (T2DM). Employing a randomized, double-blind, placebo-controlled, crossover design, this trial evaluates outcomes across glycemic indices, lipid profile, body composition, immune biomarkers, and safety parameters. Through these analyses, we sought to elucidate the functional role and translational potential of this probiotic strain in managing early-stage T2DM.

## 2. Materials and Methods

### 2.1. Tools and Materials

The probiotic used in this study was *Bifidobacterium animalis* subsp. *lactis* TISTR 2591 (BA-2591), encapsulated in 350 mg capsules. Each participant consumed three capsules daily, for a total daily dosage of 1050 mg, with a guaranteed minimum viable count of 1 × 10^9^ CFU/g. A set of validated tools and questionnaires were used to collect clinical and demographic data. A personal information questionnaire to record data on sex, age, family history of diabetes, comorbidities, anthropometric data, and blood pressure. To assess gastrointestinal tolerability, a modified version of the gastrointestinal symptom questionnaire originally developed by Bovenschen et al. (2006) was used to systematically track symptoms [11]. Blood biochemistry and body composition data were collected using standardized forms. The biochemical parameters included indicators for glycemic control, lipid profile, liver and kidney function, immune and inflammatory markers, and antioxidant capacity. Body composition measurements included body fat, BMI, visceral fat, and basal metabolic rate.

Probiotic formulation and placebo. Each capsule contained freeze-dried BA-2591 with a guaranteed viable count of 1 × 10^9^ CFU/g at manufacture, blended with food-grade excipients and filled into gelatin capsules (net fill 350 mg). Capsules were identical in appearance, smell, and taste for the probiotic and placebo. The placebo contained the same excipient blend (trehalose and maltodextrin in a 1:1 ratio) and capsule shell without live bacteria. All lots were stored at 4 °C with humidity control; independent microbiology verified the absence of viable BA-2591 in the placebo and confirmed CFU in the active product at release. The matching of excipients minimizes confounding when interpreting immune readouts (e.g., immunoglobulins) that might otherwise be affected by prebiotic carriers.

### 2.2. Study Design

This study was designed as a randomized, double-blind, placebo-controlled, crossover clinical trial to assess the safety and therapeutic efficacy of BA-2591 in individuals with T2DM. The study received ethical approval from the Human Experimentation Committee at Research Institute for Health Sciences, Chiang Mai University, under ethics code 35/65, document number 19/2023. All participants provided written informed consent before their involvement in the study.

### 2.3. Participants

Participants aged 35 to 65 years who were clinically diagnosed with T2DM within six months of enrollment, were recruited from Ban Mae Ka Subdistrict Health Promoting Hospital in Chiang Mai, Thailand, using a purposive sampling strategy. To be eligible, participants were required to be on metformin monotherapy and to have discontinued any other anti-diabetic or lipid-lowering drugs. or dietary supplements for at least four weeks before the study begun. Exclusion criteria included pregnancy or lactation, hepatic dysfunction (AST or ALT levels exceeding three times the upper limit of normal), renal impairment (estimated glomerular filtration rate below 45 mL/min or serum creatinine > 1.5 mg/dL), severe psychiatric illness, or alcohol dependence. Participants were also excluded if they showed non-compliance with study protocols or retracted their consent. The termination criteria for the study included the manifestation of severe adverse reactions to the product, two or more severe allergic reactions, or five or more significant adverse events.

### 2.4. Sample Size

The sample size was established based on previous research assessing the effects of *Gymnema inodorum* tea in individuals with T2DM, which indicated mean fasting blood glucose (FBG) levels of 141 mg/dL in the intervention group and 169 mg/dL in the control group at week eight [12]. The sample size was 17 participants per group, with a type I error rate of 5% and a power of 95%. The sample size was raised to 22 participants per group to make up for the expected 25% dropout rate. In addition, no Thai RCT of probiotics in early T2DM reported variance parameters for powering FBG at the time of design, we used a Thai trial of *Gymnema inodorum* tea targeting FBG as a pragmatic proxy for between-arm FBG differences in a comparable clinical context.

### 2.5. Intervention and Assessment

Participants were randomly assigned to two groups using a color-coded ball-draw method. Group A received the probiotic for six weeks, followed by a four-week washout period, and then a six-week placebo phase. Group B followed the inverse sequence, receiving the placebo first, followed by the washout, and then the probiotic. Baseline demographic and clinical data were collected before the intervention. Blood drawings were conducted in Day 0, Week 6, Week 10, and Week 16. Participants were instructed to fast for 10–12 h before each blood draw. Each sample consisted of 10 mL of venous blood collected in serum separator tubes and transported at controlled temperatures. Participants consumed encapsulated BA-2591 supplements daily before breakfast, and adherence was monitored through weekly phone interviews. Adverse events were systematically documented and evaluated throughout the study. All blood chemical tests, including CBC, liver and renal function tests, lipid profiles, hs-CRP, and HbA1c, were performed at the Prompt Service Center at the Faculty of Associated Medical Sciences, Chiang Mai University.

### 2.6. Outcomes Assessment

The primary outcomes of the study were changes in fasting blood glucose (FBG) levels, fasting insulin levels, and the calculated indices of insulin resistance (HOMA-IR) and pancreatic function (HOMA-β). FBG concentrations were measured using a portable glucometer (Accu-Chek Guide, Roche Diabetes Care GmbH, Mannheim, Germany), while fasting insulin levels were quantified with an enzyme-linked immunosorbent assay (ELISA) kit (Sigma-Aldrich, Cat. No. RAB0327, Darmstadt, Germany). From these values, the Homeostasis Model Assessment indices for insulin resistance (HOMA-IR) and beta-cell function (HOMA-β) were calculated [13].

Secondary outcomes included alterations in immune function markers, specifically immunoglobulin G (IgG) (Invitrogen, Cat. No. 88-50550, Vienna, Austria) and immunoglobulin M (IgM) (Invitrogen, Cat. No. 88-50620, Vienna, Austria). Adiponectin concentrations (Abcam, Cat. No. ab99968, Cambridge, UK), which reflect adipose tissue functionality, and inflammatory cytokine IL-6 (Sigma-Aldrich, Cat. No. RAB0306, Darmstadt, Germany) levels were also measured via ELISA. Additional biochemical parameters assessed were the activity of the enzyme Cathepsin D (Abcam, Cat. No. ab65302, Cambridge, UK), which measures proteolytic degradation, and the levels of malondialdehyde (MDA) via the TBARS assay [14] to indicate oxidative damage. Total antioxidant capacity was measured by the Trolox Equivalent Antioxidant Capacity (TEAC) test [15], and erythrocyte glutathione (eGSH) levels were determined using a glutathione assay kit (Sigma-Aldrich, Cat. No. CS0260, Darmstadt, Germany). At all designated time points, anthropometric data, standard hematologic profiles, and relevant biochemical markers were recorded.

### 2.7. Statistical Analysis

Descriptive statistics were used to summarize the baseline characteristics of the study participants and key parameters. The Chi-square test was employed to compare categorical variables. For continuous data, the Mann–Whitney U test was used for non-normally distributed data, while Student’s *t*-test was applied to data with a normal distribution. To evaluate intra-subject variations before and after the intervention for both the probiotic and placebo phases, the Wilcoxon signed-rank test was utilized for non-parametric data, and paired sample *t*-tests were used for normally distributed variables. All statistical analyses were performed using GraphPad Prism software version 9 (GraphPad Software, Inc., San Diego, CA, USA).

### 2.8. Use of Artificial Intelligence Tools

During the preparation of this manuscript, the authors used ChatGPT model GPT-4o (OpenAI, San Francisco, CA, USA), SciSpace version 1.5.1 (SciSpace Inc., Bengaluru, India), and QuillBot Premium version 1.2.2.0 (QuillBot, Chicago, IL, USA) (all accessed in June 2025). These tools were employed to assist with literature synthesis, sentence refinement, and scientific summarization. No AI tools were used for data collection, statistical analyses, or interpretation of study results. All AI-generated content was thoroughly reviewed and revised by the authors to ensure accuracy and appropriateness.

## 3. Results

**Figure 1:**
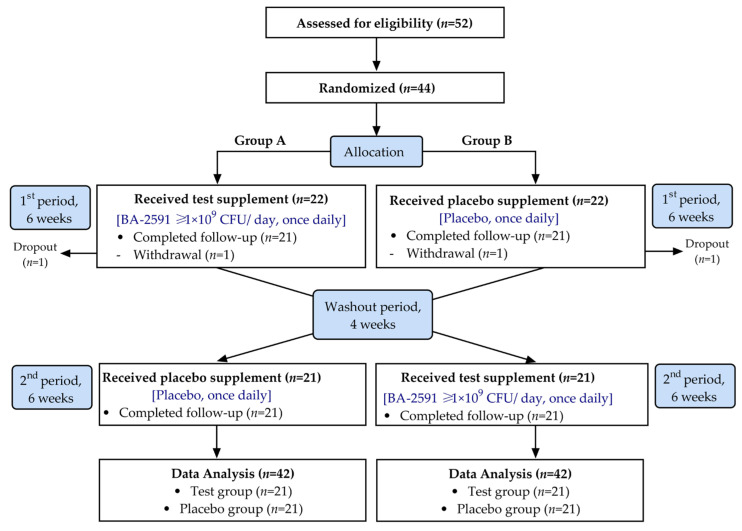
CONSORT diagram showing the study flow.

A total of 52 volunteers were initially screened for eligibility, of whom 44 met the inclusion criteria and were randomized into the study. The CONSORT diagram (Figure 1) details the study flow. These 44 participants were allocated to two groups of 22 using simple random sampling with an opaque box and colored ping-pong balls. Group A received the probiotic for six weeks, followed by a four-week washout period and a six-week placebo phase. Group B followed the inverse sequence. Two participants, one from each group, withdrew due to scheduling conflicts, resulting in 42 participants (21 per group) who completed the study and were included in the final data analysis.

### 3.1. Baseline Characteristics of Participants

The baseline characteristics of the 42 participants are summarized in Table 1. No statistically significant differences were observed between Group A and Group B for any of the assessed demographic, anthropometric, or clinical parameters (*p* > 0.05). No substantial difference was observed between the number of males and females (*p* = 0.216). The mean age of participants was 57.24 ± 5.59 years for Group A and 59.05 ± 5.88 years for Group B (*p* = 0.063). Similarly, there were no notable baseline discrepancies in metabolic parameters, such as fasting blood glucose (FBG: 135.70 ± 16.71 mg/dL for Group A vs. 134.30 ± 18.86 mg/dL for Group B, *p* = 0.320) and HbA1c (7.10 ± 1.82 for Group A vs. 6.64 ± 1.27 for Group B, *p* = 0.298). Anthropometric measurements, including Body Mass Index (BMI), waist circumference, hip circumference, and waist-to-hip ratio, were comparable between the two groups at baseline (*p* > 0.05 for all). Similarly, there are no statistically significant differences in vital signs, including systolic blood pressure (*p* = 0.193), diastolic blood pressure (*p* = 0.428), and heart rate (*p* = 0.108), at the beginning of the study.

### 3.2. Safety Evaluations

#### 3.2.1. Body Composition and Physiological Indicators

Across all four time points (Day 0, Week 6, Week 10, and Week 16), most body composition measures remained stable. The probiotic group showed a minor, non-significant increase in body weight from 58.69 ± 12.90 to 58.79 ± 12.70 kg (mean difference = 0.105 kg; 95% CI: −0.410 to 0.620; *p* = 0.202), while in the placebo group, it rose from 58.27 ± 12.39 to 58.60 ± 12.75 kg (mean difference = 0.331 kg; 95% CI: −0.184 to 0.846; *p* = 0.202). BMI, waist and hip circumferences, and waist-to-hip ratio also showed no significant changes in either group (*p* > 0.200). Systolic blood pressure in the placebo group decreased slightly from 139.14 ± 17.94 to 134.59 ± 15.09 mmHg (*p* = 0.064), while diastolic pressure and heart rate remained unchanged (*p* = 0.322 and *p* = 0.303, respectively). Fat mass slightly increased in both probiotic (29.65% ± 11.24 to 31.35% ± 610.81; *p* = 0.276) and placebo-treated group (29.98% ± 10.97 to 32.62% ± 10.00; *p* = 0.276). Visceral fat area rose marginally in the probiotic group (Δ = +0.381 cm^2^; *p* = 0.067). Bone mass showed no substantial differences, with the probiotic group changing from 2.42 ± 0.84 to 2.46 ± 0.82 kg (*p* = 0.206), and no change in the placebo group (*p* = 0.206). Notably, basal metabolic rate (BMR) declined significantly in the probiotic group (Δ = −19.520 kcal; *p* = 0.011). The incidence of gastrointestinal symptoms was low, with no significant differences between the groups. Detailed data are presented in Table 2.

#### 3.2.2. Hematological Parameters

Hematological analysis (Table 3) revealed a significant decrease in hemoglobin in the probiotic group (12.71 ± 1.57 to 12.44 ± 1.72 g/dL; *p* = 0.041). The mean corpuscular volume (MCV) decreased in the placebo group (82.72 ± 10.56 to 81.99 ± 10.70 fL; *p* = 0.003). A significant group difference in MCHC was observed (*p* < 0.001), with a reduction in the probiotic group and an increase in the placebo group. Platelet count increased in the probiotic group from 280.19 ± 86.57 × 10^3^/µL to 300.83 ± 76.99 × 10^3^/µL (*p* < 0.001), whereas it significantly decreased in the placebo group (*p* < 0.001).

#### 3.2.3. Biochemical Indicators

Biochemical analyses assessed lipid profiles (total cholesterol, triglycerides, HDL-C, LDL-C), kidney function (BUN, sCr), and liver function (AST, ALT, ALP). As shown in Table 4, the probiotic group demonstrated a significant improvement in LDL-C and the LDL-C/HDL-C ratio compared to the placebo group (*p* = 0.009 and *p* = 0.024, respectively). The placebo group exhibited a statistically significant attenuation in the rise in BUN compared to the probiotic group (*p* = 0.035). No significant effects were observed for other lipid parameters, liver enzymes (AST, ALT, ALP), or biochemical indicators.

### 3.3. Efficacy Assessment

#### 3.3.1. Effects on Changes in Fasting Blood Glucose (FBG) Levels

**Figure 2:**
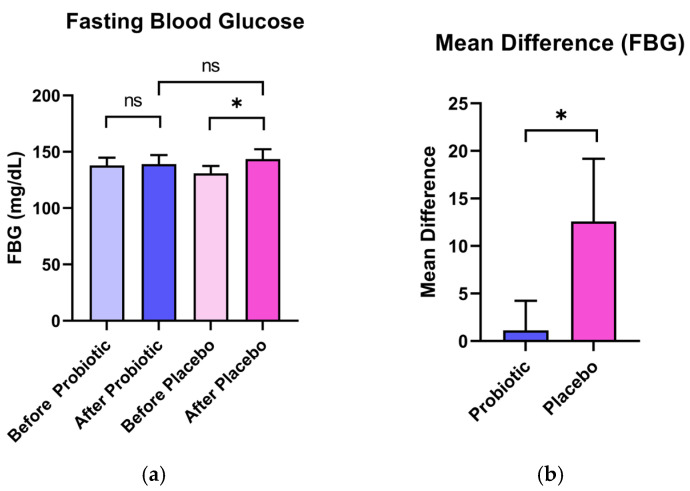
Clinical effects of a 6-week consumption of the test product on changes in fasting blood glucose levels (FBG) in volunteers (*n* = 42). (**a**) illustrates the comparison of mean values of indicators before and after consuming the test product containing the probiotic (BA-2591) and before and after consuming the placebo. (**b**) demonstrates the mean differences between volunteers in the probiotic group and those in the placebo group. ^ns^ Indicates no statistically significant difference in mean values between the two groups at *p* < 0.05. * Indicates a statistically significant difference in mean values between the two groups at *p* < 0.05.

Fasting blood glucose levels increased in both groups, but the rise was significantly attenuated in the probiotic group (137.90 ± 21.98 to 139.00 ± 25.82 mg/dL, Δ = +1.143 mg/dL) compared to the placebo group (131.00 ± 20.59 to 143.50 ± 27.76 mg/dL, Δ = + 12.570 mg/dL), with a statistically significant between-group difference (*p* < 0.001). In contrast, HbA1c levels remained stable in both groups (*p* = 0.188). These findings are detailed in Figure 2b and Table 5.

#### 3.3.2. Effects on Pancreatic Function

**Figure 3:**
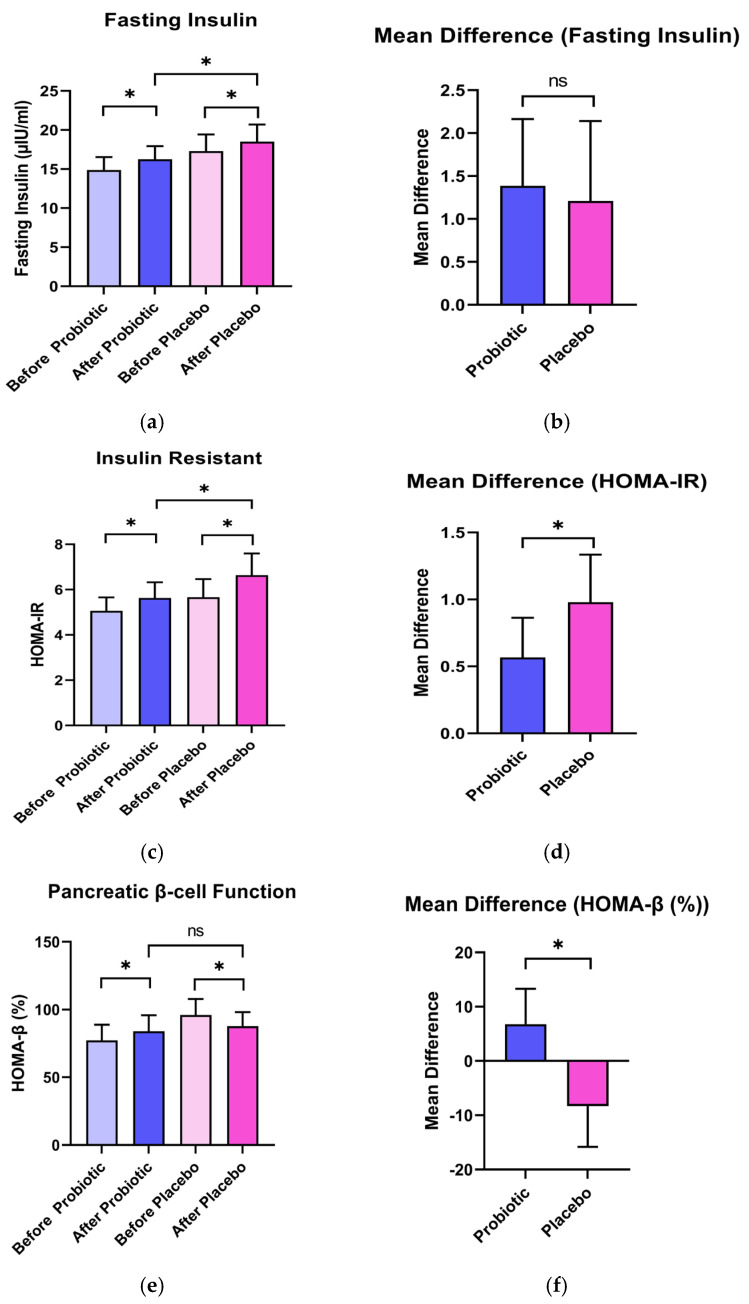
The impact of a 6-week test product consumption on pancreatic function in volunteers (*n* = 42); (**a**,**b**): Fasting insulin levels, (**c**,**d**): Insulin resistance via HOMA-IR, (**e**,**f**): Beta-cell function via HOMA-B. The findings are delineated as mean values for indicators are compared to pre- and post-consumption of the probiotic and placebo; (**a**,**c**,**e**); and mean differences between the probiotic and placebo groups are compared; (**b**,**d**,**f**). ^ns^ indicates no statistically significant difference in mean values between the two groups at *p* < 0.05. * indicates a statistically significant difference in mean values between the two groups at *p* < 0.05.

As detailed in Figure 3 and Table 5, fasting insulin levels increased in both probiotic (14.88 ± 5.28 to 16.27 ± 5.34 µIU/mL) and placebo groups (17.30 ± 6.83 to 18.51 ± 7.02 µIU/mL) without a significant intergroup difference (*p* = 0.461). However, the calculated HOMA-IR (Homeostasis Model Assessment of Insulin Resistance) which indicates the level of insulin resistance, increased to a lesser extent in the probiotic group (∆ = +0.567) compared to the placebo group (∆ = +0.980; *p* = 0.006). Furthermore, pancreatic β-cell function, as indicated by HOMA-β (Homeostasis Model Assessment of Beta-cell Function), improved significantly in the probiotic group (77.30 ± 37.05% to 84.09 ± 37.68%; Δ = +6.791) while declined in the placebo group (96.12 ± 37.64% to 87.81 ± 33.23%; Δ = −8.313), with a significant intergroup difference (*p* < 0.001).

#### 3.3.3. Effects on Immune System Response

After the intervention, immunoglobulin G (IgG) and immunoglobulin M (IgM) levels rose markedly with BA-2591 supplementation. As shown in Table 5, IgG increased by 261.5 mg/dL in the probiotic group and decreased by 250.7 mg/dL in the placebo group (*p* < 0.001). Similarly, IgM levels increased by 150.3 mg/dL in the probiotic group while declining by 131.5 mg/dL in the placebo group (*p* < 0.001).

#### 3.3.4. Effects on Adipose Tissue Function

Adiponectin is a hormone secreted by adipocytes that plays a critical role in energy metabolism, blood glucose regulation, and the modulation of inflammatory responses. It also serves as an indicator of the adipose tissue’s ability to mobilize and clear lipid accumulations. In this study, Adiponectin levels declined in both groups (probiotic: 28.61 ± 17.65 to 23.54 ± 14.09 µg/mL; placebo: 29.00 ± 16.90 to 23.99 ± 14.94 µg/mL), with no significant intergroup difference (*p* = 0.437), as shown in Table 6.

#### 3.3.5. Effects on the Protein Degradation

Cathepsin D is a lysosomal protease enzyme found in animal and human cells that plays a key role in intracellular protein degradation. In this present study, Cathepsin D activity significantly decreased in the probiotic group (32,236.00 ± 9194.00 to 30,508.00 ± 8617.00 FI units/mg; *p* = 0.005) while increasing in the placebo group, indicating significant intergroup difference. These results are presented in Table 6.

#### 3.3.6. Effects on Inflammation

High-sensitivity C-reactive protein (hs-CRP) is a well-established biomarker of systemic inflammation and is commonly used in clinical practice to assess inflammatory status and guide patient management. In this study, hs-CRP levels slightly decreased in the probiotic group (2.94 ± 3.74 to 2.89 ± 3.09 mg/L; Δ = −0.053) and increased in the placebo group (2.94 ± 3.29 to 3.20 ± 3.45 mg/L; Δ = +0.271), with no significant intergroup difference (*p* = 0.213). In addition, a pro-inflammatory cytokine, Interleukin-6 (IL-6) levels increased in both groups (probiotic: 39.99 ± 17.91 to 55.83 ± 24.12 pg/mL; placebo: 39.32 ± 16.13 to 53.31 ± 19.47 pg/mL; *p* = 0.386).

#### 3.3.7. Effects on Oxidative Stress and Antioxidant Status

Plasma malondialdehyde (MDA) levels, a biomarker of lipid peroxidation, decreased slightly in both groups without a significant intergroup difference (probiotic: 0.29 ± 0.10 to 0.25 ± 0.08 mmol/L; placebo: 0.30 ± 0.14 to 0.30 ± 0.16 mmol/L; *p* = 0.206), as shown in Table 6. Plasma total antioxidant status, assessed as Trolox Equivalent Antioxidant Capacity (TEAC), declined modestly in the probiotic group (Δ = −0.004 mg TE/mL) and increased in the placebo group (Δ = +0.002 mg TE/mL), with a significant intergroup difference (*p* < 0.001). In terms of intracellular antioxidant defense, the level of red blood cell glutathione (GSH) increased in the probiotic group (152.90 ± 65.20 to 173.50 ± 87.36 µmol/L), while slightly decreasing in the placebo group, with no significant intergroup difference (*p* = 0.190). These results are presented in Table 6.

## 4. Discussion

This randomized, double-blind, placebo-controlled crossover study investigated the physiological and metabolic impacts of a 6-week intervention with BA-2591 in middle-aged adults with T2DM. Results spanned glycemic control, immune function, oxidative stress, inflammation, adipokines, and safety indicators. A key finding of this research is the favorable safety profile of the probiotic strain, as evidenced by the absence of significant adverse gastrointestinal events and stable liver and renal function throughout the intervention periods. This aligns with previous clinical trials demonstrating the high tolerability of various *Bifidobacterium* strains in both healthy and diabetic populations [16,17,18]. Such strain-specific safety was confirmed in Thai subjects, showing no hemolysis or toxicity with BA-2591 [8]. The stability of renal markers, such as BUN and creatinine, is consistent with other studies indicating that probiotics do not impose a burden on renal function and may even support nitrogen excretion pathways [19,20].

In this study, no significant changes in body weight, BMI, or waist-to-hip ratio was observed, and metabolic improvements occurred without changes in weight or fat mass. This outcome is consistent with previous research suggesting that probiotic-driven weight changes are minimal in normal-BMI individuals over short durations [21,22]. Notably, individuals of East and Southeast Asian ancestry develop T2DM at lower BMI than Europeans and display greater visceral adiposity and earlier β-cell dysfunction at a given BMI. These ethnic/ancestral features may influence both baseline metabolic risk and the response to microbiome-directed adjuncts. Given that our cohort consisted of Thai adults with near-normal to mildly elevated BMI, the generalizability and effect size may differ from cohorts with higher adiposity [23,24]. While some specific bacterial strains like *Bifidobacterium breve* and *Lactobacillus plantarum* have been shown to reduce adiposity in overweight cohorts [25], the effects of BA-2591 appear to be more subtle. A modest, yet significant, decline in the basal metabolic rate (BMR) was noted in the probiotic group, which may reflect a shift in microbial energy harvesting or short-chain fatty acid (SCFA) profiles [26]. Notably, SCFAs such as butyrate can suppress hepatic gluconeogenesis, indirectly impacting BMR [27]. The intervention resulted in nuanced hematological changes, including a modest decrease in hemoglobin levels and a significant increase in platelet count in the probiotic group. While this may appear unexpected, certain probiotic strains modulate iron availability and uptake via lactic acid production [28]. These findings parallel to studies that link gut microbiota composition to thrombopoiesis and hematopoiesis through SCFA and IL-6 pathways [29,30]. In terms of lipid metabolism, the probiotic group showed a decrease in LDL-C and a significant improvement in the LDL-C/HDL-C ratio. This is consistent with the well-known lipid-lowering mechanism of probiotic-mediated bile salt hydrolase activity, which is particularly prominent in *Bifidobacterium* species [3,31]. The lack of significant changes in liver enzymes (AST and ALT) further supports the hepatic safety of the intervention and aligns with trials using *B. lactis* or *B. breve* in metabolic syndrome that report hepatoprotective effects without enzyme elevation [32].

The most notable finding related to efficacy was the significantly attenuated rise in fasting blood glucose (FBG) in the probiotic group, suggesting improved glycemic buffering. This result aligns with several randomized controlled trials where *Bifidobacterium* strains lowered fasting glucose by 5–10 mg/dL in diabetic and prediabetic individuals [33,34,35]. Possible mechanisms for this effect include an improved gut barrier function and the modulation of incretin hormones like GLP-1 [36]. Moreover, the between-period difference in ΔFBG (≈ 11 mg/dL) is modest compared with first-line agents such as metformin (typical HbA1c reduction ~1–2% in RCTs) but could be additive as an adjunct to stable metformin in early T2DM. Meta-analyses similarly report small, directionally favorable probiotic effects that scale with duration and strain strategy [37]. Additionally, the lesser increase in HOMA-IR in the probiotic group indicates improved insulin sensitivity. This finding is supported by the data showing that probiotics reduce endotoxemia, a driver of insulin resistance, by tightening intestinal junctions and modulating TLR-4 signaling [38,39]. The improvement in HOMA-β suggests enhanced pancreatic beta-cell responsiveness, which may be mediated by microbial anti-inflammatory effects and nutrient-sensing receptors [40]. Although fasting glucose and HOMA indices moved in favorable directions, HbA1c remained unchanged, which is expected because HbA1c integrates glycaemia over ~8–12 weeks and our treatment periods were 6 weeks with modest baseline HbA1c. Thus, phase-sensitive endpoints (FBG, HOMA-IR, and oHOMA-β) are more responsive over this timeframe, while HbA1c effects would be better evaluated in longer trials [41].

The significant increase in both IgM and IgG in the probiotic group demonstrates a strong indication of mucosal and systemic immune enhancement. This finding corroborates previous preclinical research findings [8] where BA-2591 was shown to boost IL-10 expression and serum IgG in animal model. Probiotics are also known to enhance immunity by activating dendritic cells, promoting IgA class switching, and stimulating regulatory T cells [42,43,44]. Moreover, immunoglobulin IgG/IgM increased during BA-2591, but baseline values differed across periods (Table 5); such heterogeneity in a crossover can introduce regression-to-the-mean artifacts if post-only values are compared. We therefore emphasize change-from-baseline within each period and interpret these immune findings cautiously [37].

Adiponectin, an insulin-sensitizing adipokine, declined slightly in both groups without a significant intergroup difference. Meta-analyses suggested that the probiotic modulation of adiponectin is highly dependent on the specific strain, host metabolic state, and the duration of intervention [45,46]. The 6-week duration of probiotic intervention in the present study may have been insufficient to elicit measurable effects in normoglycemic individuals with type 2 diabetes. Interestingly, cathepsin D activity decreased significantly in the probiotic group, suggesting attenuated protein degradation or stress signaling. Elevated cathepsin D has been linked to insulin resistance and adipocyte dysfunction [47], making this a promising area for future research in probiotic therapeutics.

Non-significant changes were observed for the inflammatory markers hs-CRP and IL-6, although the directional shifts favored the probiotic group. This may be attributable to previous findings that reductions in these markers are more commonly observed in individuals with elevated baseline inflammation or metabolic syndrome, probiotic anti-inflammatory effects are frequently clearer in participants with higher baseline inflammation or after longer exposure [35,48,49]. However, IL-6 may exhibit dual behavior—pro-inflammatory in chronic stress but anti-inflammatory in acute signaling—especially when induced by microbial fermentation products like lactate and SCFA [50].

Regarding the evaluation of oxidative stress markers, the probiotic group showed non-significant declines in MDA (a marker of lipid peroxidation) and a rise in GSH (cellular antioxidant compound), while slightly decreased in TEAC (plasma total antioxidant capacity) values. These are consistent with other clinical trials that have demonstrated the ability of probiotics to elevate antioxidant enzymes and GSH levels, particularly in diabetic cohorts [51,52,53]. The noted reduction in cathepsin D may also be indicative of lowered oxidative or lysosomal stress [54].

## 5. Conclusions

This randomized, double-blind, placebo-controlled crossover study evaluated the physiological and metabolic impacts of BA-2591 in adults over a 16-week period. The probiotic exhibited a favorable safety profile, with no significant gastrointestinal adverse events and stable liver and kidney function throughout the intervention periods.

The most significant metabolic outcome was a significant attenuation of fasting blood glucose elevation in the probiotic group relative to the placebo, suggesting a modest improvement in glycemic control. While the levels of HbA1c levels remained unchanged, the notable reduction in HOMA-IR and the enhancement in HOMA-β suggest improved insulin sensitivity and pancreatic β-cell function. These changes occurred independently of weight loss or BMI reduction, highlighting a possible independent mechanism of action, implying potential interactions between gut microbiota and the host.

Despite the relative stability of body composition metrics, including fat mass, waist-hip ratio, and visceral fat area, there was a notable reduction in basal metabolic rate during the probiotic phase. These observations may indicate microbial effects on energy expenditure, warranting further mechanistic exploration.

Hematological evaluations indicated a minor reduction in hemoglobin levels and a significant elevation in platelet count within the probiotic group, potentially indicating nuanced effects on hematopoiesis. A decrease in LDL-C and an enhancement in the LDL-C/HDL-C ratio were noted, aligning with the lipid-modulating characteristics ascribed to Bifidobacterium species.

The probiotic group also exhibited significant increases in serum IgM and IgG concentrations, indicating an immunostimulatory effect. Although results in the level of inflammatory markers, including hs-CRP and IL-6, did not change significantly, favorable trends were observed. Oxidative stress markers demonstrated consistent reduction in lipid peroxidation (MDA) and increases in antioxidant defense capacity (eGSH), though a slight rise in TEAC in the placebo group necessitates further investigation.

The probiotic group exhibited a notable decrease in cathepsin D activity, suggesting downregulation of lysosomal and proteolytic stress pathways— emerging biomarkers of metabolic dysfunction.

In summary, BA-2591 supplementation was safe and produced modest adjunctive benefits in fasting glycaemia, insulin dynamics, and select immune measures without changes in body composition. These data support BA-2591 as a potential adjunct to standard care in early T2DM; longer, larger, and mechanism-focused trials are required to define durability, dose–response, and effects on clinical endpoints.

## Figures and Tables

**Table 1:** Baseline characteristics of participants in the study ^a^.

Parameters	Participants (*n* = 42)		*p*-Value ^b^
	Group A (*n* = 21)	Group B (*n* = 21)	
Sex (Male/Female)	3 (14.29)/18 (85.71)	5 (23.81)/16 (76.19)	0.216
Age (years)	57.24 ± 5.59	59.05 ± 5.88	0.063
FBG (mg/dL)	135.70 ± 16.71	134.30 ± 18.86	0.320
HbA1c	7.10 ± 1.82	6.64 ± 1.27	0.298
BMI (kg/m^2^)	24.03 ± 5.26	25.19 ± 5.022	0.203
Waist cir. (cm)	83.57 ± 12.55	86.50 ± 11.12	0.210
Hip cir. (cm)	95.83 ± 10.43	95.94 ± 9.424	0.419
Waist/Hip Ratio	0.88 ± 0.06	0.90 ± 0.06	0.150
SYS (mmHg)	134.60 ± 13.40	140.40 ± 21.40	0.193
DIA (mmHg)	81.05 ± 7.27	81.33 ± 10.19	0.428
HR (BPM)	88.48 ± 8.841	86.48 ± 16.12	0.108

^a^ Values are presented as numbers (*n*, %) or mean ± standard deviation (Mean ± SD). ^b^ Qualitative data were analyzed using the Chi-square test. Quantitative data with a non-normal distribution were analyzed using the Mann–Whitney U test, while quantitative data with a normal distribution were analyzed using Student’s *t*-test.

**Table 2:** Changes in body composition and blood pressure parameters ^a^.

Parameters	Probiotic (*n* = 42)				Placebo (*n* = 42)				*p*-Value ^b^
	Before	After	Mean Difference	95% CI	Before	After	Mean Difference	95% CI	
Weight (kg)	58.69 ± 12.90	58.79 ± 12.70	0.105	−0.410 to 0.620	58.27 ± 12.39	58.60 ± 12.75	0.331	−0.184 to 0.846	0.202
BMI (kg/m^2^)	24.72 ± 5.26	24.78 ± 5.22	0.054	−0.169 to 0.276	24.54 ± 5.04	24.68 ± 5.18	0.134	−0.089 to 0.357	0.215
Hip circumference (cm)	97.22 ± 10.47	96.27 ± 9.59	−0.943	−2.44 to 0.561	96.46 ± 10.17	96.36 ± 10.08	−0.100	−1.604 to 1.404	0.216
Waist circumference (cm)	87.35 ± 12.76	86.46 ± 12.42	−0.883	−2.899 to 1.132	87.17 ± 11.56	86.28 ± 11.63	−0.891	−2.906 to 1.125	0.482
Waist/Hip Ratio	0.89 ± 0.06	0.89 ± 0.07	0.000	−0.021 to 0.0210	0.90 ± 0.06	0.89 ± 0.05	−0.009	−0.029 to 0.012	0.260
SYS (mmHg)	138.83 ± 20.29	138.57 ± 19.26	−0.262	−4.869 to 4.345	139.14 ± 17.94	134.59 ± 15.09	−4.548	−9.155 to 0.059	0.064
DIA (mmHg)	82.21 ± 9.49	80.76 ± 10.69	−1.452	−4.459 to 1.554	82.19 ± 9.54	79.64 ± 9.82	−2.548	−5.554 to 0.459	0.322
HR (BPM)	86.45 ± 12.64	86.24 ± 14.38	−0.214	−2.820 to 2.391	87.69 ± 13.78	86.45 ± 13.81	−1.238	−3.843 to 1.367	0.303
Fat mass (%)	29.65 ± 11.24	31.35 ± 10.81	1.702	−1.072 to 4.477	29.98 ± 10.97	32.62 ± 10.00	2.648	−0.126 to 5.422	0.276
Body water (%)	52.16 ± 5.76	51.65 ± 5.69	−0.505	−1.007 to −0.002	51.82 ± 5.62	51.58 ± 5.33	−0.233	−0.735 to 0.269	0.136
Visceral fat (cm^2^)	7.63 ± 3.45	8.01 ± 3.15	0.381	−0.159 to 0.921	7.86 ± 3.08	7.90 ± 3.22	0.036	−0.504 to 0.576	0.067
Bone mass (kg)	2.42 ± 0.84	2.46 ± 0.82	0.045	−0.085 to 0.174	2.42 ± 0.78	2.42 ± 0.84	−0.000	−0.130 to 0.130	0.206
BMR (kcal)	1127.97 ± 187.28	1108.44 ± 180.97	−19.520	−38.490 to −0.556	1103.09 ± 174.19	1108.19 ± 183.84	5.095	−13.87 to 24.06	0.011 *

^a^ The values are presented as mean ± standard deviation (Mean ± SD), *n* = 42. ^b^ The *p*-value indicates the comparison of the mean difference in indicators before and after probiotic consumption with the mean difference in indicators before and after placebo consumption; Quantitative data with non-normal distribution were analyzed using the Wilcoxon signed-rank test, while quantitative data with normal distribution were analyzed using the paired sample *t*-test. * (*p* < 0.05) indicates a statistically significant difference between the two groups of participants.

**Table 3:** Changes in hematological parameters ^a^.

Parameters	Probiotic (*n* = 42)				Placebo (*n* = 42)				*p*-Value ^b^
	Before	After	Mean Difference	95% CI	Before	After	Mean Difference	95% CI	
WBC (×10^3^ µL)	7.98 ± 2.18	7.88 ± 2.58	−0.103	−0.606 to 0.399	8.15 ± 2.08	7.74 ± 2.08	−0.404	−0.907 to 0.098	0.084
RBC (×10^6^ µL)	4.74 ± 0.67	4.69 ± 0.76	−0.047	−0.242 to 0.1474	4.79 ± 0.77	4.79 ± 0.64	−0.007	−0.201 to 0.188	0.140
HBG (g/dL)	12.71 ± 1.57	12.44 ± 1.72	−0.271	−0.768 to 0.225	12.82 ± 2.19	12.74 ± 1.71	−0.076	−0.572 to 0.420	0.041 *
HCT (%)	38.69 ± 4.06	38.24 ± 4.82	−0.452	−2.017 to 1.112	39.32 ± 6.22	38.87 ± 4.48	−0.450	−2.015 to 1.115	0.488
MCV (fl)	82.62 ± 10.79	82.66 ± 11.17	0.033	−0.870 to 0.937	82.72 ± 10.56	81.99 ± 10.70	−0.733	−1.637 to 0.170	0.003 *
MCH (pg)	27.22 ± 4.37	26.96 ± 4.39	−0.252	−0.608 to 0.104	27.03 ± 4.29	26.94 ± 4.34	−0.098	−0.454 to 0.258	0.138
MCHC (g/dL)	32.80 ± 1.30	32.50 ± 1.27	−0.300	−0.502 to −0.098	32.54 ± 1.36	32.71 ± 1.36	0.174	−0.028 to 0.376	<0.001 *
PLT count (×10^3^ µL)	280.19 ± 86.57	300.83 ± 76.99	20.640	5.036 to 36.250	301.28 ± 74.33	292.54 ± 89.84	−8.738	−24.34 to 6.868	<0.001 *
Neutrophil (%)	57.05 ± 8.12	56.02 ± 8.77	−1.024	−3.895 to 1.847	57.45 ± 8.12	55.05 ± 10.11	−2.405	−5.276 to 0.466	0.089
Lymphocyte (%)	32.24 ± 7.35	33.00 ± 6.53	0.762	−1.820 to 3.344	31.83 ± 6.32	33.74 ± 8.38	1.905	−0.677 to 4.487	0.070
Monocyte (%)	6.83 ± 2.04	6.74 ± 2.15	−0.095	−0.645 to 0.454	6.78 ± 1.93	7.16 ± 2.36	0.381	−0.169 to 0.931	0.085
Eosinophil (%)	3.57 ± 3.12	3.71 ± 3.74	0.143	−0.506 to 0.791	3.62 ± 3.14	3.81 ± 3.40	0.191	−0.458 to 0.838	0.448
Basophil (%)	0.54 ± 0.55	0.62 ± 0.62	0.071	−0.194 to 0.337	0.50 ± 0.55	0.54 ± 0.55	0.048	−0.218 to 0.314	0.381

^a^ The values are presented as mean ± standard deviation (Mean ± SD), *n* = 42. ^b^ The *p*-value indicates the comparison of the mean difference in indicators before and after probiotic consumption with the mean difference in indicators before and after placebo consumption; Quantitative data with non-normal distribution were analyzed using the Wilcoxon signed-rank test, while quantitative data with normal distribution were analyzed using the paired sample *t*-test. * (*p* < 0.05) indicates a statistically significant difference between the two groups of participants.

**Table 4:** Changes in biochemical blood indicators ^a^.

Parameters	Probiotic (*n* = 42)				Placebo (*n* = 42)				*p*-Value ^b^
	Before	After	Mean Difference	95% CI	Before	After	Mean Difference	95% CI	
BUN (mg/dL)	14.44 ± 3.91	14.35 ± 4.32	−0.086	−1.436 to 1.264	14.47 ± 3.91	15.38 ± 4.36	0.917	−0.433 to 2.267	0.035 *
Creatinine (mg/dL)	0.82 ± 0.24	0.82 ± 0.22	−0.003	−0.038 to 0.032	0.85 ± 0.26	0.84 ± 0.26	−0.014	−0.049 to 0.021	0.177
Cholesterol (mg/dL)	189.50 ± 44.35	186.40 ± 39.19	−3.095	−11.15 to 4.963	188.45 ± 46.56	190.48 ± 48.40	2.024	−6.035 to 10.080	0.077
Triglyceride (mg/dL)	172.47 ± 73.62	170.62 ± 88.62	−1.857	−32.980 to 29.260	177.16 ± 94.57	189.16 ± 102.22	12.000	−19.120 to 43.120	0.215
HDL-C (mg/dL)	55.52 ± 19.16	55.83 ± 20.26	0.310	−3.659 to 4.278	56.17 ± 26.00	54.36 ± 20.68	−1.810	−5.778 to 2.159	0.213
LDL-C (mg/dL)	102.21 ± 37.68	99.17 ± 36.08	−3.048	−11.590 to 5.494	99.64 ± 38.59	104.88 ± 38.78	5.238	−3.304 to 13.780	0.009 *
LDL-c/HDL-c	1.98 ± 0.79	1.95 ± 0.78	−0.036	−0.196 to 0.124	1.98 ± 0.85	2.06 ± 0.80	0.089	−0.071 to 0.248	0.024 *
AST/SGOT (U/L)	36.45 ± 23.33	36.88 ± 26.08	0.429	−3.955 to 4.812	34.67 ± 21.64	37.50 ± 29.07	2.833	−1.550 to 7.217	0.212
ALT/SGPT (U/L)	30.24 ± 24.82	32.88 ± 29.86	2.643	−1.069 to 6.354	30.90 ± 28.53	31.19 ± 27.86	0.286	−3.426 to 3.997	0.170
ALP (U/L)	90.86 ± 31.14	90.07 ± 32.63	−0.786	−6.280 to 4.708	90.43 ± 30.68	88.00 ± 27.28	−2.429	−7.922 to 3.065	0.298

^a^ The values are presented as mean ± standard deviation (Mean ± SD), *n* = 42. ^b^ The *p*-value indicates the comparison of the mean difference in indicators before and after probiotic consumption with the mean difference in indicators before and after placebo consumption; Quantitative data with non-normal distribution were analyzed using the Wilcoxon signed-rank test, while quantitative data with normal distribution were analyzed using the paired sample *t*-test. * (*p* < 0.05) indicates a statistically significant difference between the two groups of participants.

**Table 5:** Changes in blood glucose levels, pancreatic function, and immune response ^a^.

Parameters	Probiotic (*n* = 42)				Placebo (*n* = 42)				*p*-Value ^b^
	Before	After	Mean Difference	95% CI	Before	After	Mean Difference	95% CI	
**Blood Glucose Level**									
FBG (mg/dL)	137.90 ± 21.98	139.00 ± 25.82	1.143	−7.399 to 9.685	131.00 ± 20.59	143.50 ± 27.76	12.570	4.030 to 21.110	<0.001 *
HbA1c (%)	6.88 ± 1.62	6.95 ± 1.52	0.071	−0.2073 to 0.350	6.84 ± 1.35	6.86 ± 1.41	0.014	−0.264 to 0.292	0.188
**Pancreatic Beta Cell Function**									
Fasting Insulin (µIU/mL)	14.88 ± 5.28	16.27 ± 5.34	1.386	−0.894 to 3.666	17.30 ± 6.83	18.51 ± 7.02	1.208	−1.072 to 3.488	0.461
HOMA-IR	5.06 ± 1.90	5.63 ± 2.22	0.567	−0.284 to 1.417	5.66 ± 2.56	6.64 ± 3.06	0.980	0.129 to 1.830	0.006 *
HOMA-β (%)	77.30 ± 37.05	84.09 ± 37.68	6.791	−7.913 to 21.490	96.12 ± 37.64	87.81 ± 33.23	−8.313	−23.020 to 6.391	<0.001 *
**Immune Response**									
IgM (mg/dL)	330.60 ± 218.50	480.90 ± 342.80	150.300	57.690 to 242.90	553.00 ± 364.40	421.50 ± 246.30	−131.500	−224.100 to −38.880	<0.001 *
IgG (mg/dL)	479.40 ± 233.70	740.90 ± 256.70	261.500	130.50 to 392.40	638.40 ± 362.20	387.7 ± 164.30	−250.700	−381.700 to −119.700	<0.001 *

^a^ The values are presented as mean ± standard deviation (Mean ± SD), *n* = 42. ^b^ The *p*-value indicates the comparison of the mean difference in indicators before and after probiotic consumption with the mean difference in indicators before and after placebo consumption; quantitative data with non-normal distribution were analyzed using the Wilcoxon signed-rank test, while quantitative data with normal distribution were analyzed using the paired sample *t*-test. * (*p* < 0.05) indicates a statistically significant difference between the two groups of participants.

**Table 6:** Changes in cellular protein degradation, inflammation, oxidative stress, and antioxidant capacity ^a^.

Parameters	Probiotic (*n* = 42)				Placebo (*n* = 42)				*p*-Value ^b^
	Before	After	Mean Difference	95% CI	Before	After	Mean Difference	95% CI	
**Adipocyte function**									
Adiponectin (µg/mL)	28.61 ± 17.65	23.54 ± 14.09	−5.075	−9.704 to −0.446	29.00 ± 16.90	23.99 ± 14.94	−5.005	−9.634 to −0.375	0.437
**Protein Degradation**									
Cathepsin D (FI unit/mg protein)	32,236 ± 9194	30,508 ± 8617	−1728.0	−4992 to 1536	30,795 ± 8660	32,529 ± 8291	1734.0	−1530 to 4998	0.005 *
**Inflammation**									
hs-CRP (mg/L)	2.94 ± 3.74	2.89 ± 3.09	−0.053	−0.810 to 0.704	2.94 ± 3.29	3.20 ± 3.45	0.271	−0.486 to 1.020	0.213
IL−6 (pg/mL)	39.99 ± 17.91	55.83 ± 24.12	15.840	6.486 to 25.190	39.32 ± 16.13	53.31 ± 19.47	13.990	5.409 to 22.570	0.386
**Lipid peroxidation**									
Plasma MDA (mmol/L)	0.29 ± 0.10	0.25 ± 0.08	−0.041	−0.112 to 0.030	0.38 ± 0.14	0.30 ± 0.16	−0.069	−0.140 to 0.001	0.206
**Antioxidant**									
TEAC (mg TE/mL)	0.200 ± 0.005	0.196 ± 0.005	−0.004	−0.006 to −0.001	0.199 ± 0.005	0.202 ± 0.005	0.002	−0.001 to 0.005	<0.001 *
RBCs Glutathione (µmol/L)	152.90 ± 65.20	173.50 ± 87.36	20.600	−27.940 to 69.130	193.60 ± 104.60	194.60 ± 95.65	0.933	−47.600 to 49.470	0.190

^a^ The values are presented as mean ± standard deviation (Mean ± SD), *n* = 42. ^b^ The *p*-value indicates the comparison of the mean difference in indicators before and after probiotic consumption with the mean difference in indicators before and after placebo consumption; Quantitative data with non-normal distribution were analyzed using the Wilcoxon signed-rank test, while quantitative data with normal distribution were analyzed using the paired sample *t*-test. * (*p* < 0.05) indicates a statistically significant difference between the two groups of participants.

## Data Availability

Due to the anonymization of participants’ names and information to ensure privacy and comply with research ethics regulations, which require respect for participants’ rights and well-being, personal data of individual participants cannot be disclosed and must be kept confidential.

## Abbreviations

The following abbreviations are used in this manuscript:

BA-2591	*Bifidobacterium animalis* subsp. *lactis* TISTR 2591
T2DM	Type 2 diabetes mellitus
FBG	Fasting Blood Glucose
HOMA-IR	Homeostasis Model Assessment Indices for Insulin Resistance
HOMA-β	Homeostasis Model Assessment Indices for Beta-cell Function
CFU	Colony Forming Unit
CONSORT	Consolidated Standards of Reporting Trials
